# Retroflexion of the Uterus

**Published:** 1902-06-14

**Authors:** 


### HOSPITAL CLINICS AND MEDICAL PROGRESS.

### RETROFLEXION OF THE UTERUS.

THERE are few conditions which have led to more acrimonious discussions among medical practitioners than those which, whether they be "flexions" or "versions," may be grouped together as backward displacements of the uterus. Such discussions have had to do not with treatment alone, the pathology and the etiology of these cases have also been brought in question, while it has even been held by some that as a cause of the symptoms which have been attributed to it, retroflexion of the uterus is a condition which may be entirely neglected. As to treatment the discussion has raged with greatest acerbity around the question of the pessary, and the shape which this instrument ought to take. Meanwhile, there has existed a curious lack of exact knowledge as to the conditions of the parts. Perhaps this was inevitable so long as pathology and morbid anatomy could only be studied by aid of post-mortem examinations. Modern surgery, however, has done much for pathology by enabling us to see during life the actual state of the parts complained of, and so far as backward displacement of the uterus is concerned, the result of investigation by way of abdominal section has been to show that while in many cases the diagnosis by vaginal examination had been wrong, the mass taken for a retroflexed fundus being in reality an ovary, or a tube, or a tumour, in other cases a pessary which was imagined to be holding the uterus in position was doing nothing of the sort; all that the instrument was doing being to hold the organ a little higher than usual in the pelvis.

Nevertheless, if one is to look at the matter impartially, one must find some explanation of the enormous popularity among women of the various forms of pessaries. Mr. Bland Sutton recently stated at the Polyclinic that from inquiries he had made from several instrument makers he had found that one London maker supplied about 10,000 rubber rings and 3,000 Hodges in a year, while another was putting upon the market about 36,000 rings of various patterns per annum, and that vast quantities of pessaries were imported from Germany. It is probable enough that a large proportion of these instruments are lying unused in the surgeries of practitioners up and down the country, but their continued production in such numbers shows at least that a very large number of women have recourse to such things. What these instruments do is to relieve some of the discomfort arising from the slighter degrees of prolapse of the uterus, and one can hardly doubt that in many of the cases of retroflexion in which pessaries give relief it is by their action on the accompanying prolapse that this relief is obtained; that is by holding the uterus up rather than by holding it straight. We will not go so far as Mr. Bland Sutton—who appears to hold that there is only one proper place for pessaries designed to cure retroflexion, namely the fire—for there is evidence, and, after all, one must go by evidence, that pessaries in some cases do good. We may, however, without injustice express the opinion that the ingenious inventors of these strangely-shaped instruments while aiming at one thing—the retroflexion—have in a considerable number of cases hit something else, namely, the accompanying prolapse, and have so given relief.

By looking into the pelvis from above Mr. Bland Sutton has shown that in a considerable number of cases, believed to be cases of retroflexion, the symptoms are due to small ovarian cysts, or dermoids, or to chronically inflamed tubes or small pedunculated fibroids. Moreover, he has been able, by operating from above in cases which have been presumed to have been reduced, and in which the pessaries which were imagined to be holding the uterus in position were still *in situ*, to show that the flexion did, as a fact, remain unreduced; and he has come to the conclusion that in inveterate cases in which a retroflexed uterus is a source of suffering and demands active treatment, the most satisfactory manner of affording relief is by performing the operation of hysteropexy—sewing the fundus of the uterus to the abdominal wall. He especially insists on the important fact that in certain cases the pain and distress accompanying retroflexion of the uterus are not due to the flexion itself but to the position of the ovaries in relation to the uterus, for when one or both of these with their accompanying portion of tube happen to fall into the recto-vaginal pouch and thus to lie beneath the body of the uterus, they are rubbed and pressed upon in a very uncomfortable manner, which is but increased if a pessary be introduced in such a way as to press upon the ovary or the tube. In regard to the operation of hysteropexy, it is said to be one of the simplest that can be performed on any of the abdominal viscera.

